# Cincinnati Changes

**Published:** 1867-09

**Authors:** 


***Cincinnati Changes.***

Prof. E. S. Conner has resigned his position in the Cincinnati Medical School, to accept the Chair of Medical Chemistry and Physics in the Ohio Medical College.

Dr. D. S. Young has been appointed Professor of Surgery in the Cincinnati Medical College. Our correspondent in Cincinnati speaks of both these gentlemen in complimentary terms.

